# The Role of Sarcopenia and Myosteatosis in Short- and Long-Term Outcomes Following Curative-Intent Surgery for Hepatocellular Carcinoma in a European Cohort

**DOI:** 10.3390/cancers14030720

**Published:** 2022-01-30

**Authors:** Franziska Alexandra Meister, Georg Lurje, Suekran Verhoeven, Georg Wiltberger, Lara Heij, Wen-Jia Liu, Decan Jiang, Philipp Bruners, Sven Arke Lang, Tom Florian Ulmer, Ulf Peter Neumann, Jan Bednarsch, Zoltan Czigany

**Affiliations:** 1 Department of Surgery and Transplantation, Faculty of Medicine, University Hospital RWTH Aachen, 52074 Aachen, Germany; fmeister@ukaachen.de (F.A.M.); suekran.verhoeven@rwth-aachen.de (S.V.); gwiltberger@ukaachen.de (G.W.); lheij@ukaachen.de (L.H.); wliu@ukaachen.de (W.-J.L.); djiang@ukaachen.de (D.J.); svlang@ukaachen.de (S.A.L.); fulmer@ukaachen.de (T.F.U.); uneumann@ukaachen.de (U.P.N.); jbednarsch@ukaachen.de (J.B.); 2 Department of Surgery, Campus Charité Mitte|Campus Virchow-Klinikum, Charité-Universitätsmedizin, 13353 Berlin, Germany; georg.lurje@charite.de; 3 Institute of Radiology, Faculty of Medicine, University Hospital RWTH Aachen, 52074 Aachen, Germany; pbruners@ukaachen.de; 4 Department of Surgery, Maastricht University Medical Centre (MUMC), 6229 Maastricht, The Netherlands

**Keywords:** HCC, body composition, myosteatosis, sarcopenia, liver resection

## Abstract

**Simple Summary:**

Recent studies have shown that pathological changes of body composition, in particular reduced muscle mass (sarcopenia) and impaired muscle quality (myosteatosis), are linked to poor outcomes in a variety of clinical conditions. Hepatocellular carcinoma (HCC) is the most frequent primary malignant tumor of the liver in the Western hemisphere and remains a prominent cause of cancer-associated mortality. The present study investigates the prognostic value of alterations in body composition in predicting perioperative morbidity, mortality and long-term oncological outcome in HCC using preoperative computed-tomography-based segmentation. Our study found supporting evidence for the relevance of muscle quality over quantity in a European population and verifies the predictive role of myosteatosis in patients suffering from HCC, with a particularly significant value in the earlier perioperative phase.

**Abstract:**

Alterations of body composition, especially decreased muscle mass (sarcopenia) and impaired muscle quality (myosteatosis), are associated with inferior outcomes in various clinical conditions. The data of 100 consecutive patients who underwent partial hepatectomy for hepatocellular carcinoma (HCC) at a German university medical centre were retrospectively analysed (May 2008–December 2019). Myosteatosis and sarcopenia were evaluated using preoperative computed-tomography-based segmentation. We investigated the predictive role of alterations in body composition on perioperative morbidity, mortality and long-term oncological outcome. Myosteatotic patients were significantly inferior in terms of major postoperative complications (Clavien–Dindo ≥ 3b; 25% vs. 5%, *p* = 0.007), and myosteatosis could be confirmed as an independent risk factor for perioperative morbidity in multivariate analysis (odds ratio: 6.184, confidence interval: 1.184–32.305, *p* = 0.031). Both sarcopenic and myosteatotic patients received more intraoperative blood transfusions (1.6 ± 22 vs. 0.3 ± 1 units, *p* = 0.000; 1.4 ± 2.1 vs. 0.3 ± 0.8 units, respectively, *p* = 0.002). In terms of long-term overall and recurrence-free survival, no statistically significant differences could be found between the groups, although survival was tendentially worse in patients with reduced muscle density (median survival: 41 vs. 60 months, *p* = 0.223). This study confirms the prognostic role of myosteatosis in patients suffering from HCC with a particularly strong value in the perioperative phase and supports the role of muscle quality over quantity in this setting. Further studies are warranted to validate these findings.

## 1. Introduction

Body composition (BC) naturally varies among individuals depending on various factors, such as age and sex [1]. The assessment of BC has recently gained increased interest in a variety of pathological conditions, and generalized deterioration of BC is frequently observed in critically ill patients and patients suffering from various types of cancer [2,3,4,5,6,7]. While the loss of muscle mass and function, defined as sarcopenia and myosteatosis describes qualitative changes in muscle structure with increased intra- and intermyocellular fat accumulation [8,9,10,11,12].

Hepatocellular carcinoma (HCC) has an ever-increasing global incidence and is a major contributor to cancer-associated mortality worldwide [13]. Most patients suffering from HCC have an underlying chronic liver disease and are often affected by metabolic comorbidities at the time of diagnosis [14]. This is especially true in the present era with a rapidly increasing incidence of comorbidities, including metabolic syndrome, obesity, type II diabetes and non-alcoholic fatty liver disease (NAFLD) [13]. Due to their comorbidities and the underlying chronic liver disease, HCC patients are often unfit for curative surgical therapy. Those who can undergo partial hepatectomy have an increased risk of perioperative morbidity and poor long-term outcomes [15,16].

Recent high-impact studies by our group have reported a high prevalence of sarcopenia and myosteatosis and a strong association of BC with adverse perioperative outcomes in patients suffering from chronic liver disease and undergoing liver transplantation [3,17]. In line with this, Harimoto et al. detected sarcopenia as a predictor for worse overall survival (OS) in a Japanese cohort undergoing partial hepatectomy for HCC. Similar results have been reported by others over the recent years [18,19]. Concerning myosteatosis, Kaibori et al. reported a strong association of fat infiltration in skeletal muscle with inferior overall survival in patients suffering from HCC [20]. Although there are multiple studies on the association of pathological BC patterns and inferior outcomes in HCC, these patient cohorts are usually highly heterogeneous, utilizing a wide range of treatments and/or predominantly focusing on sarcopenia [21,22]. Western data on the short- and long-term prognostic role of the emerging factor myosteatosis following curative-intent surgery for HCC are still lacking.

Based on our recent findings in liver transplantation cohorts which received much scientific interest within the community [3,17,23], in this study, we aimed to comprehensively assess the predictive value of BC, including sarcopenia and myosteatosis in short- and long-term outcomes in a Western European single-centre cohort of patients suffering from HCC and undergoing liver resection with curative intent.

## 2. Materials and Methods

### 2.1. Patients, Ethics, and Eligibility

All consecutive patients undergoing partial hepatectomy for HCC with a curative intent between May 2008–December 2019 at the University Hospital RWTH Aachen (UH-RWTH), Aachen, Germany, were considered for inclusion into this retrospective analysis. Clinical staging was performed prior to the surgical indication, and patients with systemic or irresectable disease were rolled out. Patients where abdominal staging was performed using MRI were *per se* not eligible for the utilized segmentation analysis and therefore have been excluded.

The present study was conducted following the principles of the current version of the Declaration of Helsinki and the good clinical practice (ICH-GCP) guidelines. It was approved by the RWTH-Aachen University Institutional Review Board (EK 115/20 and EK 341/21).

### 2.2. Image Analysis and Segmentation

**Figure 1:**
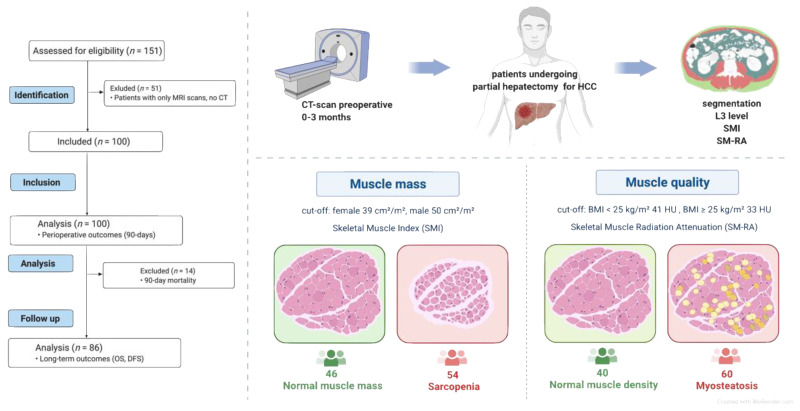
Study design. Patients undergoing curative-intent surgery for HCC were analysed. Sarcopenic and myosteatotic patients were identified using CT-based segmentation at the level of the third lumbar vertebra. This figure was created with BioRender.com. Abbreviations used: MRI: magnetic resonance imaging; OS: overall survival; DFS: disease-free survival; CT: computed tomography; HCC: hepatocellular carcinoma; L3: third lumbar vertebrae; BMI: body mass index; HU: hounsfield units; SMI: skeletal muscle index; SM-RA: skeletal muscle radiation attenuation.

Computed tomography imaging was carried out at the UH-RWTH Aachen up to 12 weeks prior to surgery (Figure 1). Imaging data were analysed in a semi-automatic fashion by the same investigator who was blinded for the remaining clinical data and outcomes of the patients, as described before by our group [3]. Briefly, at the level of the third lumbar vertebra (L3), a single cross-sectional image was analysed using the 3D Slicer software platform version 4.1 and BC module (https://www.slicer.org/, 1 January 2022) [24]. Skeletal muscle index (SMI) and skeletal muscle radiation attenuation (SM-RA) are widely used parameters to characterize muscle mass (sarcopenia) and myosteatosis. Established cutoffs for patients suffering from chronic liver disease were used to identify patients at risk (SMI: female 39 cm^2^/m^2^, male 50 cm^2^/m^2^; SM-RA < 41 HU for patients with a body mass index (BMI) up to 24.9 kg/m^2^ and <33 HU for patients with a BMI ≥ 25 kg/m^2^) (Figure 1) [25,26].

### 2.3. Clinical Data Collection and Patient Follow Up

Data were obtained from a prospectively maintained institutional database and analysed retrospectively. The indication for the operative procedure for each patient was made by at least one experienced hepatobiliary surgeon and was approved by the institutional interdisciplinary tumor board. The partial hepatectomy was performed either laparoscopically or conventionally based on an individual case-by-case decision considering the difficulty of the procedure and other risk factors as well as patient preferences [27]. Techniques of partial hepatectomy, including the method of parenchyma dissection, were described by our group in previous studies [15,28,29]. The outpatient clinic of the UH-RWTH Aachen, as well as the local community-based hepatologist network, provided the follow-up data used in this study.

Scores and classifications reported in this study have been described by our group and by others in previous reports (including ASA classification (American Society of Anesthesiologists), ALBI grade (Albumin-Bilirubin) [30], labMELD (model for end-stage liver disease), Charlson Comorbidity Index (CCi) [31]; Clavien–Dindo classification (CD) and the Comprehensive Complication Index (CCI) [32,33], procedural costs [34], calculation of transfusion and the length of hospitalisation [35,36]).

### 2.4. Statistical Analysis

The primary endpoint of this study was defined as the incidence of perioperative in-hospital major morbidity (defined by CD ≥ 3b) [32]. Overall perioperative outcome, length of hospitalization, 90-day mortality, 60-month overall survival and 60-month disease-free survival were analysed and reported as secondary endpoints. Continuous data were reported as mean ± standard deviation and absolute and relative frequencies in the case of categorical variables. For the analysis of categorial data, Chi-squared tests and Fisher’s exact test were used where appropriate. Continuous data were analysed using Student’s *t* test, the Mann–Whitney U test, and the Kruskal–Wallis H test, as applicable.

The ability of BC parameters to predict perioperative outcomes was assessed using uni- and multivariable logistic regression analyses. The overall and disease-free survival curves were analyzed by the Kaplan–Meier method and compared with the log-rank test. Patients deceased within 90 days after the surgical procedure have been excluded from all survival analyses.

The level of statistical significance was set to *p* < 0.05. Statistical analysis was performed using SPSS Statistics 24 (IBM Corp., Armonk, NY, USA).

## 3. Results

### 3.1. Study Population Characteristics

Within the study period, a total of 151 patients underwent partial hepatectomy for HCC with curative intent at our institution. Some 51 had insufficient preoperative CT imaging (MRI scans) and were not included in the analysis (Figure 1). Finally, 100 patients with a mean age of 67 ± 11 were included. In the analysed cohort, 28 patients (28%) were female. The mean labMELD was 8 ± 3, and 42 patients had liver cirrhosis confirmed by histology. In total, 67 patients were categorized as performance status ASA III or higher, and 17 patients underwent preoperative therapy including portal venous embolization (PVE, 6 patients), systemic therapy with sorafenib (1 patient), transarterial radioembolization (TARE, 3 patients) and transarterial chemoembolization (TACE, 7 patients), respectively (Table 1). The mean largest tumor diameter was 72 ± 41 mm, and 71 patients suffered from HCC belonging to UICC category I or II (Union for International Cancer Control) (36/35).

Microvascular invasion was pathologically confirmed in 52 cases (52%), and most patients had G2 tumors (77%). The underlying liver disease was non-alcoholic fatty liver disease (NAFLD) in 38% of cases, followed by alcoholic (28%) and viral disease (25%). The most common operative procedures were hemihepatecomy (25%) and bisegmentectomy (25%), followed by atypical non-anatomical resections (24%). A laparoscopic approach was used in 21% of cases, and R0 resection was achieved in 85% of the patients, respectively (Table 1).

### 3.2. Body Composition

The median time between CT scan used for analysis and the operative procedure was 19 [6,7,8,9,10,11,12,13,14,15,16,17,18,19,20,21,22,23,24,25,26,27,28,29,30,31,32,33,34,35,36,37,38,39,40,41,42,43,44,45,46,47] days. BMI was 26 ± 4 with a mean SMI of 45 ± 9 cm^2^/m^2^ for all included patients. Mean SMI was 37 ± 5 cm^2^/m^2^ for females and 49 ± 8 cm^2^/m^2^ for males, respectively (Table 1). The mean overall SM-RA was 33 ± 10 HU, 34 ± 11 HU for females and 32 ± 10 HU for male patients (Table 1). A total of 54 patients were categorized as sarcopenic, and 60 patients suffered from myosteatosis according to our pre-defined cutoff values.

Patient characteristics were in general equally distributed between patients with myosteatosis and those with normal muscle density with slight differences. Patients with mysteatosis had a significantly lower serum albumin (36 ± 11 g/L vs. 43 ± 5 g/L, *p* = 0.001, Table 1) and suffered more often from alcoholic liver disease (38% vs. 13%, *p* = 0.005, Table 1), while viral liver disease was observed less frequently (14% vs. 42%, *p* = 0.001, Table 1). In the myosteatotic subgroup, the frequency of preoperative PVE was lower (2% vs. 13%, *p* = 0.025, Table 1).

Characteristics in sarcopenic and non-sarcopenic patients were equally distributed, with the exception of BMI, which was significantly higher in patients without sarcopenia (24 ± 3 kg/m^2^ vs. 29 ± 4 g/L, *p* = 0.000, Table 1). Sarcopenic patients suffered more frequently from alcoholic liver disease than non-sarcopenic patients (13% vs. 2%, *p* = 0.025, Table 1), and the type of surgery also differed slightly in the two groups. Detailed demographics are displayed in Table 1.

### 3.3. Myosteatosis, Sarcopenia and Their Value in Predicting Perioperative Outcomes

A total of 17% (17 out of 100) of all patients showed major (CD ≥ 3b) postoperative complications during their hospital stay following partial hepatectomy according to the Clavien–Dindo classification (Table 2 and Table 3). The distribution of major morbidity is depicted in Table 2. For the analysed cohort, the mean CCI score was 21 ± 89, and the mean length of hospital stay was 14 ± 13 days (Table 2). The overall 90-day mortality was 14 out of 100 (14%). The causes of 90-day mortality are depicted in detail in Table 2.

A mean of 1 ± 1.8 RBC and 2 ± 2.7 FFP units were administered during the initial surgery (Table 3). Mean estimated costs were EUR 13.4 ± 7.6 thousand (TEuro). Significantly more patients in the myosteatosis group developed major complications compared to patients with normal muscle density (25% vs. 5%, *p* = 0.007, Table 2). CCI was slightly higher in myosteatotic patients, but the difference was not significant (24 ± 32 vs. 17 ± 24, *p* = 0.689, Table 3). Similar, estimated procedural costs were slightly increased in mysteototic patients 12.2 ± 5.9 vs. 14.3 ± 8.5 TEuro, *p* = 0.383, Table 3) without statistical significance. Regarding 90-day mortality, no significant difference was observed (18% vs. 8%, *p* = 0.125). In line with the above-described findings, the mean length of hospital stay was two days longer in the myosteatosis group but showed no significant difference (15 ± 15 vs. 13 ± 10 days, *p* = 0.380, Table 3). Intraoperatively transfused RBC units were significantly higher in myosteatotic patients (1.4 ± 2.1 vs. 0.3 ± 0.8 units, *p* = 0.002, Table 3), whereas the number of FFP units administered was comparable in both groups (2.2 ± 2.8 vs. 1.6 ± 2.5 units, *p* = 0.263, Table 3).

Similar but slightly less prominent observations have been made between the subgroups of patients with and without sarcopenia regarding major complications (15% vs. 20%, *p* = 0.529, Table 3), CCI (20 ± 26 vs. 23 ± 32, *p* = 0.724, Table 3), cost estimation (14 ± 8.4 vs. 13 ± 6.8, TEuro *p* = 0.724, Table 3), 90-day mortality (17% vs. 11%, *p* = 0.437, Table 3) and length of hospital stay (15 ± 14 vs. 14 ± 13 days, *p* = 0.560, Table 2). However, similar to myosteatosis, sarcopenia was also associated with an increased need for intraoperative RBC transfusions (1.6 ± 22 vs. 0.3 ± 1 units, *p* = 0.000, Table 3).

In the multivariable analysis, male sex (OR 10.477 95%CI 1.210–90.705, *p* = 0.033), duration of the operative procedure ≥ 210 min (OR 5.385 95%CI 1.476–19.650, *p* = 0.011) and myosteatosis (SM-RA: OR 6.184 95%CI 1.184–32.305, *p* = 0.031) have been identified as independent predictors of major morbidity following partial hepatectomy for HCC (Table 4).

### 3.4. Overall and Disease-Free Survival

**Figure 2:**
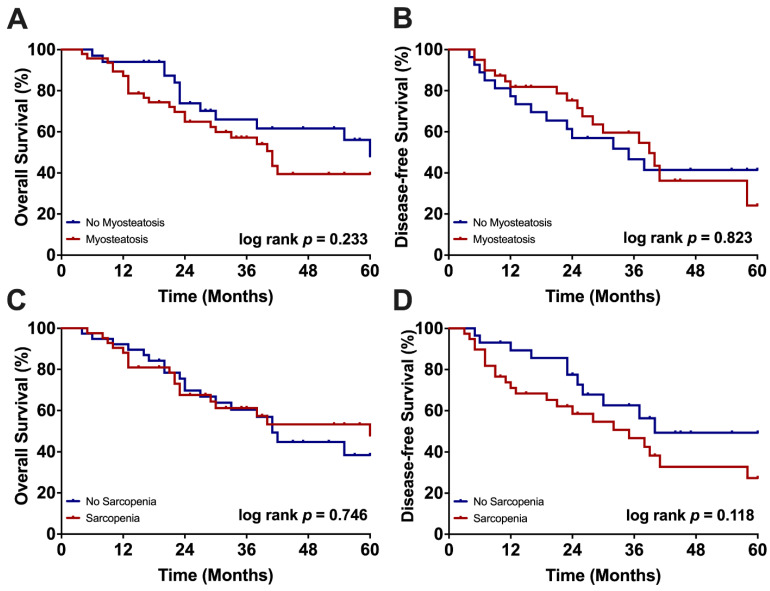
Overall and disease-free survival stratified by myosteatosis and sarcopenia. (**A**) Five-year survival of myosteatotic and non-myosteatotic patients. (**B**) Recurrence-free survival of myosteatotic and non-myosteatotic patients. (**C**) Five-year survival of sarcopenic and non-sarcopenic patients. (**D**) Recurrence-free survival of sarcopenic and non-sarcopenic patients.

The median follow-up period for the included patients was 52 months, with a median OS of 42 months and a median DFS of 37 months. Alterations of muscle quality and muscle mass seemingly had no statistically significant effects on long-term overall and recurrence-free survival in our analysis. It should be noted, however, that even though the differences did not reach the level of statistical significance, survival curves for OS showed different characteristics in myosteatotic and non-myosteatotic patients, with longer median survival in the absence of pathological muscle fat (median survival: 41 vs. 60 months; 1 year: 87% vs. 100%, 3 years: 57% vs. 66%, 5 years: 39% vs. 48%, *p* = 0.223; Figure 2). Interestingly, DFS was inferior, even though not statistically significant, in sarcopenic patients (median DFS: 35 vs. 40 months; 1-year: 71% vs. 89%, 3-years: 47% vs. 63%, 5-years: 27% vs. 49%, *p* = 0.118; Figure 2).

## 4. Discussion

The present study provides new insights into the understanding of the prognostic role of pathological alterations in the skeletal muscle compartment in HCC patients undergoing partial hepatectomy. Pathological alterations of BC were highly prevalent in our cohort, with 60% and 54% of our patients being myosteatotic and sarcopenic, respectively. While myosteatosis showed a significant negative effect on early morbidity, sarcopenia failed to stratify our patients into various risk groups based on short- or long-term outcomes.

Hepatocellular carcinoma is frequently associated with advanced parenchymal liver disease and cirrhosis [37]. Previous evidence from basic and clinical research has demonstrated a strong correlation between chronic liver disease (CLD) and pathological alterations of BC [38]. Even though the complex mechanisms of liver-muscle cross-talk are not completely understood, it has been shown that not only can CLD can trigger muscle wasting and changes in skeletal muscle structure, but also, the skeletal muscle compartment can contribute to the further progression of liver disease [26,38]. Due to this strong bidirectional association between the liver and skeletal muscle compartment, changes in BC have received a strongly increasing attention from the scientific community as potentially modifiable risk factors for inferior outcomes in patients with liver disease [38,39]. Although previous studies have shown that malnutrition, frailty, the loss of muscle mass and function are associated with higher rates of complications and inferior outcomes in various patient cohorts [3,5,17,23,40], there is only limited evidence available comparing the prognostic effects of qualitative changes of the skeletal muscle seen in myosteatosis versus qualitative muscle loss (sarcopenia) in a homogeneous cohort of HCC patients undergoing curative-intent surgery.

In this retrospective, single-centre study, we show that myosteatosis is superior to sarcopenia in predicting major complications including in-hospital mortality following curative-intent surgery in HCC patients. While mysoteatosis was significantly associated with a higher incidence of major postoperative complications (*p* = 0.007) and an increased need for intraoperative blood transfusion (*p* = 0.002), sarcopenia has failed to stratify our patients based on morbidity in our group analysis. Myosteatosis was also identified as an independent predictor of major morbidity in our multivariate logistic regression analysis. It should be noted, however, that the effects of body composition were rather modest, as further factors of perioperative outcomes, including morbidity assessed by CCI and length of hospital stay, were slightly inferior in myosteatotic patients but showed no statistical significance (Table 2). Further, no significant differences were observed in terms of long-term oncological outcomes, even though the overall survival curve of the non-myosteatotic patients still showed favorable characteristics compared to the myosteatotic sub-cohort. The rather modest nature of our findings and between-group differences might be associated with the limited sample-size of our cohort, especially when compared to Asian studies [21].

One of the early landmark studies by Harimoto et al. from Japan reported significantly worse OS in the presence of sarcopenia in an Asian cohort of 186 patients who underwent partial hepatectomy for HCC [18]. This study did not report SM-RA as a parameter of myosteatosis. Furthermore, following the seminal report of Harimoto, other also predominantly Asian studies delivered controversial findings on the prognostic effects of sarcopenia in HCC patients [18,41,42,43,44,45].

A meta-analysis by Xu et al. included 6 studies with a total of 1420 patients and concluded that sarcopenia might be associated with adverse outcomes [21]. Interestingly, this meta-analysis has also described an association between the presence of preoperative sarcopenia and larger diameter tumors [21]. This was in line with our findings as both our mysteatotic and sarcopenic sub-cohorts have presented with larger tumors, even though the difference did not reach the level of statistical significance.

The molecular mechanisms leading to sarcopenia and myosteatosis are still the subject of ongoing basic and translational research. While mysteatosis may be observed both in normal weight and underweight patients, it can be assumed that skeletal muscle serves as more than just an ectopic energy storage site and that mechanisms other than excessive fat consumption may play a role in the development of pathological myosteatosis and fat deposition [38,46]. In our cohort, myosteatotic patients showed a significantly lower amount of serum albumin prior to surgery; however, the BMI was similar in both groups. This observation may support the hypothesis that restricted liver function and disease-related malnutrition trigger muscle wasting and quantitative alterations of the skeletal muscle compartment [38]. There was an increasing awareness for malnutrition and BC over the past years which is well-demonstrated by the fact that new international consensus guidelines were appearing recently for the first time on nutrition and BC assessment in CLD and in surgical as well as liver transplant candidates [39,47]. Nevertheless, the clinical translation of these recommendations is still limited and considerably delayed in many centres, which underlines the importance of generating more quality clinical evidence.

The findings of the current study should be interpreted in light of potential limitations. Recently, the importance of BC assessment has been recognized by top-notch international associations and expert groups [39,48,49,50], which led to a general recommendation regarding the measurement of muscle alterations in patients with liver disease. In these recent guidelines, imaging-based BC assessment is recognized as the gold-standard technique anytime a cross-sectional scan is available. However, several major limitations remain, such as the large heterogeneity of the literature in terms of diagnostic criteria, assessment approach and cutoff values. Therefore, in this study, we used the most widely recommended cutoffs for SMI and SM-RA [3,48]. Nevertheless, multiple other muscle area-, volume-, and thickness-based indices and also a large number of other cutoff values for different muscle compartments exist and have been reported by others, which limits the comparability of our findings with some other reports [6,51,52].

Due to its retrospective nature, this study did not include any functional analysis of patient frailty, fitness and muscle strength, which should also be noted as a significant limitation. It should be noted as well that in this cohort, a relatively high (14%) 90-day mortality was reported. This observation can be at least partially explained by the high proportion of patients with significant comorbidities with high ASA grades, extensive tumor burden and extended resections. Further, our final dataset was relatively small compared to some other studies, especially from Asian centres, which might partially explain the observed rather modest between-group differences.

## 5. Conclusions

Notwithstanding some obvious limitations described above, this report is one of the first comprehensive studies to evaluate and compare the prognostic value of both myosteatosis and sarcopenia in predicting short- and long-term outcomes following partial hepatectomy for HCC in a European single-centre cohort. The findings support the prognostic role of nutritional/BC screening in patients undergoing curative-intent oncological liver surgery. Body composition assessment represents an excellent tool which can easily be integrated into the preoperative clinical assessment and decision making and could help identify patients with higher malnutritional risk who could particularly benefit from various “prehabilitation” or “BC upstaging” approaches in terms of exercise, nutritional changes and pharmacological interventions [53]. Further prospective clinical trials with larger sample sizes are warranted to extend and validate our findings.

## Figures and Tables

**Table 1:** Patient characteristics.

Characteristics	All Patients	Myosteatosis		Sarcopenia		*p*-Value
	*n* = 100	no *n* = 40	yes *n* = 60	no *n* = 46	yes *n* = 54	
Patient age (years)	67 ± 11	64 ± 14	70 ± 8	66 ± 11	68 ± 11	0.056/0.354
Patient BMI	26 ± 4	26 ± 3	26 ± 5	29 ± 4	24 ± 3	0.822/**0.000**
Patient sex ratio (F:M)	28:72	10 (25):30 (75)	18 (30):42 (70)	10 (22):36 (78)	18 (33):36 (67)	0.585/0.198
ASA						
1	2	1 (3)	1 (2)	0	2 (4)	0.771/0.187
2	33	19 (47)	14 (23)	17 (37)	16 (30)	**0.012**/0.437
3	59	19 (47)	40 (67)	26 (56)	33 (61)	0.056/0.462
4	6	1 (3)	5 (8)	3 (7)	3 (6)	0.229/0.839
Patient CCi	6.2 ± 1.5	5.8 ± 1.6	6.4 ± 1.3	6.1 ± 1.4	6.2 ± 1.6	0.079/0.716
Preoperative labMELD	8 ± 3	7 ± 1	9 ± 3	8 ± 3	8 ± 2	0.126/0.509
Milan criteria	22	12 (30)	10 (17)	14 (30)	8 (15)	0.089/0.069
Cirrhosis	42	17 (43)	25 (42)	20 (44)	22 (41)	0.990/0.710
Liver disease						
Alcoholic	28	5 (13)	23 (38)	8 (17)	20 (37)	**0.005**/**0.029**
NAFLD	38	14 (35)	24 (40)	20 (43)	18 (33)	0.614/0.298
Viral	25	17 (42)	8 (14)	15 (33)	10 (18)	**0.001**/0.105
Other	9	4 (10)	5 (8)	3 (7)	6 (12)	0.775/0.424
Preoperative Hb (g/dL)	13.2 ± 1.6	13.3 ± 2	13.1 ± 1.2	13.3 ± 1.9	13.1 ± 1.3	0.180/0.141
Preoperative PT (%)	92.4 ± 14.7	95 ± 12.9	90.8 ± 15.6	91.9 ± 14.9	92.8 ± 14.8	0.122/0.868
Preoperative AFP (µg/L)	2400 ± 9735	2693 ± 12006	2186 ± 7921	2655 ± 10359	2047 ± 9039	0.172/0.316
Preoperative Platelets (G/l)	247 ± 119	239 ± 103	251 ± 129	231 ± 91	259 ± 138	0.766/0.534
Preoperative AST (U/L)	54 ± 37	49 ± 28	57 ± 40	59 ± 38	51 ± 35	0.618/0.362
Preoperative ALT (U/L)	47 ± 46	45 ± 42	48 ± 49	46 ± 43	48 ± 49	0.680/0.673
Preoperative GGT (U/L)	170 ± 164	134 ± 135	193 ± 177	157 ± 170	181 ± 159	0.060/0.260
Preoperative Albumin (g/L)	38 ± 10	43 ± 5	36 ± 11	38 ± 9	38 ± 10	**0.001**/0.794
ALBI						
Grade 1	73	33 (83)	40 (66)	31 (68)	42 (77)	0.081/0.244
Grade 2	24	7 (17)	17 (29)	14 (30)	10 (19)	0.214/0.164
Grade 3	3	0	3 (5)	1 (2)	2 (4)	0.151/0.655
SMI (cm^2^/m^2^)						
All	45 ± 9	48 ± 8	46 ± 10	53 ± 7	40 ± 6	0.079/**0.000**
Female	37 ± 5	39 ± 4	36 ± 5	42 ± 2	34 ± 4	0.191/**0.000**
Male	49 ± 8	51 ± 6	48 ± 9	56 ± 4	43 ± 5	0.209/**0.000**
SM-RA (HU)						
All	33 ± 10	42 ± 5	27 ± 7	33 ± 10	32 ± 10	**0.000**/0.561
Female	34 ± 11	46 ± 5	28 ± 6	35 ± 13	34 ± 9	**0.000**/0.869
Male	32 ± 10	40 ± 5	26 ± 7	33 ± 9	31 ± 10	**0.000**/0.424
Preoperative Therapy						
PVE	6	5 (13)	1 (2)	4 (9)	2 (4)	**0.025**/0.295
Sorafenib	1	0	1 (2)	0	1 (2)	0.412/0.354
TACE	7	4 (10)	3 (5)	4 (10)	3 (5)	0.337/0.540
TARE	3	0	3 (5)	1 (2)	2 (4)	0.151/0.655
Surgical Procedure						
Atypical/Non-Anatomic.	24	10 (25)	14 (23)	14 (30)	10 (19)	0.848/0.164
Segmentectomy	20	9 (22)	11 (18)	9 (20)	11 (20)	0.610/0.920
Bisegementectomy	6	1 (3)	5 (8)	3 (7)	3 (6)	0.229/0.839
Hemihepatectomy	25	10 (25)	15 (25)	11 (24)	14 (26)	1.000/0.817
Extended resection	25	7 (17)	13 (22)	4 (9)	16 (29)	0.610/**0.009**
ALPPS	2	1 (3)	1 (2)	2 (4)	0	0.771/0.122
Other	3	2 (5)	1 (2)	3 (6)	0	0.338/0.057
Laparoscopic Procedure	21	9 (22)	12 (20)	9 (20)	12 (22)	0.836/0.788
Tumor Stage UICC						
I	36	16 (40)	20 (33)	18 (39)	17 (31)	0.669/0.424
II	35	14 (35)	21 (35)	16 (35)	19 (35)	1.000/0.966
IIIa	18	6 (15)	12 (20)	8 (18)	10 (19)	0.524/0.884
IIIb	5	1 (2.5)	4 (7)	2 (4)	3 (6)	0.349/0.782
IIIc	2	1 (2.5)	1 (2)	0	2 (3)	0.771/0.187
IVa	3	1 (2.5)	2 (3)	2 (4)	1 (2)	0.811/0.466
IVb	1	1 (2.5)	0	0	1 (2)	0.218/0.354
Tumor Grading						
G1	4	2 (5)	2 (3)	3 (6)	1 (2)	0.677/0.235
G2	77	30 (75)	47 (78)	34 (74)	43 (80)	0.698/0.498
G3	19	8 (20)	11 (19)	9 (20)	10 (18)	0.835/0.894
Microvascular Invasion	52	18 (45)	34 (57)	22 (48)	30 (56)	0.149/0.476
Largest Tumor Diameter (mm)	72 ± 41	64 ± 30	77 ± 48	64 ± 35	79 ± 45	0.317/0.067
Number of Tumors	1.9 ± 1.3	1.8 ± 1.5	1.9 ± 1.3	1.7 ± 1.3	2 ± 1.4	0.268/0.076
R0 Resection	85	34 (85)	51 (85)	41 (89)	44 (82)	0.840/0.384

Values were given as mean ± standard deviation or numbers and (percent). Abbreviations used: BMI: body mass index; ASA: American Society of Anesthesiologists; MELD: model for end-stage liver disease; AFP: alphafetoprotein; AST: aspartate aminotransferase; ALT: alanine aminotransferase; CCi: Charlson Comorbidity Index; NAFLD: non-alcoholic fatty liver disease; GGT: gamma glutamyltransferase; SMI: skeletal muscle index; SM-RA (HU): skeletal muscle radiation attenuation Hounsfield units; PVE: portal venous embolization; TACE: transarterial chemoembolization; TARE: transarterial radioembolization; ALPPS: associating liver partition with portal vein ligation for staged hepatectomy; UICC: Union for International Cancer Control.

**Table 2:** Major morbidity and 90-day mortality.

	In-Hospital Morbidity CD ≥ 3b & (CD5) ^1^	90-Day Mortality after Discharge & (in Total)
Sepsis	10 (5)	1 (6)
Cardiac/pulmonary	4 (0)	1 (1)
Hemorrhage	1 (1)	0 (1)
Post-hepatectomy liver failure	2 (2)	0 (2)
Domestic death/reason unknown	not applicable	4 (4)
Total	17 (8)	6 (14)

^1^ Refers to Clavien et al. [32]; abbreviations used: CD: Clavien–Dindo classification.

**Table 3:** Perioperative outcome stratified by body composition.

	All Patients	No	Yes	*p*-Value
*Myosteatosis*	*n* = 100	*n* = 40	*n* = 60	
≥CD3b morbidity ^1^ *n* (%)	17 (17)	2 (5)	15 (25)	**0.007**
Hospital stay (days)	14 ± 13	13 ± 10	15 ± 15	0.380
Intraoperative RBC transfusion (units)	1 ± 1.8	0.3 ± 0.8	1.4 ± 2.1	**0.002**
Intraoperative FFP transfusion (units)	2 ± 2.7	1.6 ± 2.5	2.3 ± 2.8	0.263
CCI ^2^	21 ± 89	17 ± 24	24 ± 32	0.689
Cost estimation (TEuro) ^3^	13.4 ± 7.6	12.2 ± 5.9	14.3 ± 8.5	0.383
*Sarcopenia*	*n* = 100	*n* = 46	*n* = 54	
≥CD3b morbidity *n* (%)	17 (17)	9 (20)	8 (15)	0.529
Hospital stay (days)	14 ± 13	14 ± 13	15 ± 14	0.560
Intraoperative RBC transfusion (units)	1 ± 1.8	0.3 ± 1	1.6 ± 2.2	**0.000**
Intraoperative FFP transfusion (units)	2 ± 2.7	1.6 ± 2.2	2.4 ± 3	0.341
CCI	21 ± 89	23 ± 32	20 ± 26	0.724
Cost estimation (TEuro)	13.4 ± 7.6	14 ± 8.4	13 ± 6.8	0.626

^1^ Refers to Clavien et al. [32] ^2^ Refers to Slankamenac et al. [33] ^3^ Refers to Staiger et al. [34]. Abbreviations used: CD: Clavien-Dindo classification, ICU: intensive care unit, RBC: red blood cell units, FFP: fresh frozen plasma units, CCI: Comprehensive Complication Index, TEuro: thousand Euros.

**Table 4:** Uni- and multivariable logistic regression analysis for 90-day major morbidity (Clavien–Dindo ≥ 3b).

			Univariable Analysis		Multivariable Analysis	
	Major Complications (CD ≥ 3b) ^1^ *n* = 17	No/Minor Complications (CD0-3a) ^1^ *n* = 83	Odds-Ratio (95% Confidence Interval)	*p* Value	Odds-Ratio (95% Confidence Interval)	*p* Value
Age ≥ 65 years	12 (71)	53 (64)	1.358 (0.437–4.228)	0.597		
BMI ≥ 25	16 (94)	48 (58)	0.820 (0.288–2.338)	0.711		
Sex Male	16 (94)	56 (68)	**7.714 (0.972–61.246)**	**0.053**	**10.477 (1.210–90.705)**	**0.033**
ASA ≥ 3	12 (71)	53 (64)	1.358 (0.437–4.228)	0.598		
Cirrhosis yes	10 (59)	32 (39)	2.232 (0.771–6.462	0.139		
Milan yes	2 (12)	20 (24)	2.281 (0.473–11.000)	0.304		
Preoperative albumin cutoff 40	11 (65)	31 (37)	**3.075 (1.034–9.143)**	**0.043**	2.767 (0.763–10.033)	0.122
Largest tumor diameter ≥ 50 mm	13 (77)	52 (63)	1.750 (0.521–5.875)	0.365		
Multinodular tumor yes	7 (41)	31 (37)	1.084 (0.373–3.148)	0.882		
Macrovascular invasion yes	5 (29)	18 (22)	1.296 (0.402–4.179)	0.664		
Preoperative TACE yes	1 (6)	6 (7)	0.802 (0.090–7.127)	0.834		
Preoperative PVE yes	1 (6)	5 (6)	0.975 (0.107–8.918)	0.982		
Intraoperative FFP yes	10 (59)	33 (40)	2.176 (0.723–6.496)	0.166		
Intraoperative RBC yes	7 (41)	25 (30)	1.804 (0.605–5.385)	0.290		
Extended Resection yes	2 (12)	18 (22)	0.481 (0.101–2.303)	0.360		
Duration Surgery ≥ 210 min	12 (71)	35 (42)	**3.291 (1.063–10.195)**	**0.039**	**5.385 (1.476–19.650)**	**0.011**
Laparoscopic procedure	1 (6)	20 (24)	0.194 (0.024–1.554)	0.122		
Sarcopenia (SMI) Yes	8 (47)	46 (55)	0.715 (0.251–2.035)	0.530		
Myosteatosis (SM-RA) Yes	15 (88)	45 (54)	**6.333 (1.361–29.463)**	**0.019**	**6.184 (1.184–32.305)**	**0.031**

Values were given as mean ± standard deviation or numbers and (percent). Results of the logistic regression were given as odds ratios with 95% confidence interval. ^1^ Refers to Clavien et al. [32] Abbreviations used: BMI: Body mass index; ASA: American Society of Anesthesiologists; TACE: transarterial chemoembolization; PVE: portal venous embolization; FFP: fresh frozen plasma; RBC: red blood cell unit; SMI: skeletal muscle index; SM-RA (HU): skeletal muscle radiation attenuation Hounsfield units.

## Data Availability

All relevant data were reported within the manuscript. Further supporting data will be provided upon written request addressed to the corresponding author.
